# Involvement of Cholinergic Dysfunction and Oxidative Damage in the Effects of Simulated Weightlessness on Learning and Memory in Rats

**DOI:** 10.1155/2018/2547532

**Published:** 2018-02-08

**Authors:** Yongliang Zhang, Qiong Wang, Hailong Chen, Xinmin Liu, Ke Lv, Tingmei Wang, Yanli Wang, Guohua Ji, Hongqing Cao, Guanghan Kan, Yinghui Li, Lina Qu

**Affiliations:** ^1^School of Life Sciences, Northwestern Polytechnical University, Xi'an 710072, China; ^2^The Affiliated (TCM) Hospital/School of Pharmacy/Sino-Portugal TCM International Cooperation Center, Southwest Medical University, Luzhou 646000, China; ^3^State Key Laboratory of Space Medicine Fundamentals and Application, China Astronaut Research and Training Center, Beijing 100094, China; ^4^Institute of Medicinal Plant Development, Chinese Academy of Medical Sciences and Peking Union Medical College, Beijing 100193, China

## Abstract

The present study aimed to determine how the learning and memory gradually change with the prolonged hindlimb unloading (HU) treatment in rats. Different HU durations (7 d, 14 d, 21 d, and 28 d) in Sprague-Dawley (SD) rats were implemented. Cognitive function was assessed using the Morris water maze (MWM) and the shuttle box test. Additionally, parameters about cholinergic activity and oxidative stress were tested. Results showed that longer-than-14 d HU led to the inferior performances in the behavioral tasks. Besides, acetylcholine esterase (AChE) activity, malondialdehyde (MDA) level in brain, reactive oxygen species (ROS), and 8-hydroxy-2-deoxyguanosine (8-OHdG) concentrations of HU rats were significantly increased. Furthermore, choline acetyltransferase (ChAT), superoxide dismutase (SOD), and catalase (CAT) activity in brain were notably attenuated. Most of these effects were more pronounced after longer exposure (21 d and 28 d) to HU, although some indicators had their own characteristics of change. These results indicate that cholinergic dysfunction and oxidative damage were involved in the learning and memory impairments induced by longer-than-14 d HU. Moreover, the negative effects of HU tend to be augmented as the HU duration becomes longer. The results may be helpful to present possible biochemical targets for countermeasures development regarding the memory deficits under extreme environmental conditions.

## 1. Introduction

Microgravity, as a basic environmental factor in spaceflight, can influence the physiological conditions as well as psychological functions. Numerous studies were focused on the function of osteomuscular, immune, and cardiovascular systems in weightless environment [1–3]. Reports about nervous system function are relatively less, notably cognition. Only few studies have evaluated cognitive function during space travel or parabolic flights. But the results are not consistent, some of which are even contradictory. A few studies reported no effects [4, 5] or enhancement in cognitive function [6], whereas other results proved that cognitive function showed a certain degree of decline during the weightlessness [7–9]. The discrepancies might mostly be due to differences in exposure duration to microgravity. Thus, a definite and deep understanding of the effects of microgravity on cognitive function is required as a predisposition to design efficient countermeasures to minimize the negative impact on human performance. Given the complexity of spaceflight missions and numerous related technical problems with data acquisition, ground-based models play a significant role in space exploration. Hindlimb unloading (HU) with −30°angle and head-down bed rest with −6°angle are the two major models for studying the effects of microgravity on rat/mouse and human, respectively. These models can cause cephalad redistribution of body fluid, which is similar to that seen in microgravity [10]. However, most of the ground-based simulation experiments were carried out only for one certain period; long-term and dynamic effects of simulated weightlessness have not been reported. A relatively long and continuous dynamic study would be a good solution.

Previous studies have revealed that increased lipid peroxidation and oxidative stress were observed during or after spaceflights, and these effects were more pronounced after long-duration spaceflights [11, 12]. Moreover, growing evidence shows that the activation of the oxidative stress may cause lipid peroxidation, reduced antioxidant enzyme activity, and increased DNA damage, which are all related to cognitive decline [13, 14]. And administration of antioxidant agents could improve such deficits [15, 16]. In addition, the central cholinergic system plays a pivotal role in learning and memory process and has been the center of attention regarding diseases characterized by a cognitive deficit [17, 18]. These findings strongly suggest that cholinergic dysfunction and oxidative stress are closely associated with cognitive impairment. It was reported that malondialdehyde (MDA) was increased in rats that were tail-suspended for 3 weeks to simulate microgravity [19]. For 7-day simulated weightlessness mice, reactive oxygen species (ROS) increased in brain stem and frontal cortex, and it was accompanied by increase of lipid peroxidation in different brain regions [20]. Nevertheless, the role of cholinergic system in memory under simulated microgravity and the alterations of oxidative stress status with the extended HU treatment need further study.

Considering all the aforementioned, we implemented HU for four different duration in rats and then evaluated the cognitive ability at the different time points. Furthermore, some parameters about cholinergic function and oxidative stress were simultaneously detected with behavioral experiments. All these were meant to clarify the HU effects on learning and memory in rats and investigate the role of cholinergic function and oxidative damage in this process.

## 2. Materials and Methods

### 2.1. Animals

Male Sprague-Dawley (SD) rats (200–220 g) were purchased from the Laboratory Animal Institute of the Chinese Academy of Medical Sciences (Beijing, China). They were housed in groups of 5 animals per cage under a 12 h/12 h light/dark cycle at constant temperature (22 ± 2°C) and humidity (50 ± 10%). All animals had free access to standard diet and sterilized drinking water. The experimental procedures were in accordance with institutional animal care guidelines and were approved by the local institutional committee. Every effort was made to minimize the number and suffering of the animals used. All behavioral experiments were carried out between 9:00 am and 6:00 pm.

### 2.2. Experimental Procedures

**Figure 1:**
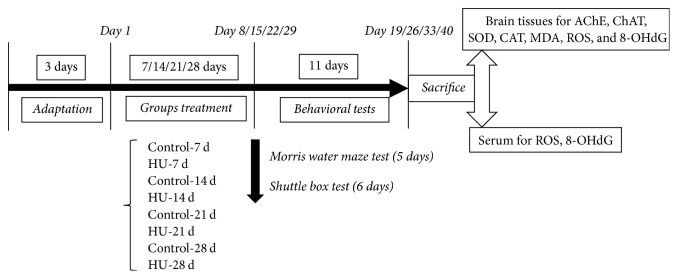
A schematic diagram of experimental design, behavioral studies, and biochemical testing. HU: hindlimb unloading; AChE: acetylcholine esterase; ChAT: choline acetyltransferase; SOD: superoxide dismutase; CAT: catalase; MDA: malondialdehyde; ROS: reactive oxygen species; 8-OHdG: 8-hydroxy-2-deoxyguanosine.

After 3 days of adaptation, rats were randomly divided into eight groups: Control-7 d group, HU-7 d group, Control-14 d group, HU-14 d group, Control-21 d group, HU-21 d group, Control-28 d group, and HU-28 d group (*n* = 10 for each group). The experimental procedure was illustrated in Figure 1. For each rat, the testing time of each day costs no more than 30 min.

### 2.3. HU Treatment

HU was performed by tail suspension [21]. The cage for tail suspension was a 26 cm × 26 cm × 30 cm plexiglass box with a crossbar. Briefly, adhesive sponge tape strips along the width of the tail were adhered laterally along the two sides of the proximal two-thirds of the tail. These longitudinal strips were then secured to the tail by 1 cm wide medical tape strips wrapped circumferentially along the length of the tail. The rats were suspended via a small chain which was preattached to the crossbar at the top of the cage. Adjustments to the length of the chain were made as necessary to prevent the hindlimbs of rats from touching any supportive surfaces while the forelimbs maintained contact with the cage floor. The animals were maintained in a 30° head-down tilt. The control animals were maintained in the same environment as HU rats, but were not tail-suspended.

### 2.4. Morris Water Maze (MWM) Test

Spatial memory acquisition and retention abilities of rats were evaluated by using MWM test [22]. In brief, the maze was a black circular tank filled with 24 ± 1°C water. An imaginary “+” divided the tank into four equal quadrants. Black ink was used to make the water opaque. A black platform (diameter 9 cm; height 24 cm) submerged 1 cm below the surface of the water was positioned in the middle of one of the quadrants, which provided the only escape from water. Each rat underwent three successive trials a day for 4 days in memory acquisition trials (training). The sequence of water entering positions differed daily, but the location of the platform was constant. Latency to find the platform was measured up to a maximum of 90 s. After locating the platform, the rat was left there for 15 s prior to the next trial. If the rat failed within 90 s, it was guided to the platform and allowed to stay there for 15 s. Latency was recorded for each trial. The interval between the trials was no more than 60 s. On the fifth day, a probe test was performed to measure the strength of spatial memory retention, during which rats were allowed to swim freely for 90 s in the pool without platform. There were two indexes calculated: the number of times when rats exactly crossed over the previous position of the platform (number of target crossings) and the distance spent in the target quadrant.

### 2.5. Shuttle Box Test

The two-way avoidance was applied and the procedures in [23] were followed with some modification. Test was performed in a shuttle box (70 × 70 × 70 cm), which consists of two similar compartments equipped with independent electrifiable grid floors, separated by a plank with a square hole (10 × 10 cm) in the center. Prior to this session, rats were allowed to explore the apparatus and to be familiar with the learning environment for 5 min. In each trial, a blue light (10 Lux) was switched on alternately in the two compartments and used as the conditioned stimulus (CS). The CS was kept for 5 s, followed by the unconditioned stimulus (UCS, 0.5 mA foot shock) for a maximum of 30 s. If the rat moved to the other side of the box during the period of CS, there was no shock and the response was scored as an active avoidance. If the rat did not cross to the other side during the first 5 s of the CS, a foot shock was delivered until the rat escaped or until 30 s had elapsed. If the rat crossed while the shock was being presented, the response was scored as a passive avoidance. A CS followed by an UCS is considered as a trial. The avoidance training sessions consisted of 30 trials with 10 s intertrial period. The training schedule was controlled by a computer that scored the number of active avoidances and escape responses (passive avoidances), which reflects the learning performance of the animals. The behavioral assay lasted 6 days, with one session per day.

### 2.6. Preparation of Brain Tissues and the Serum Samples

After the behavior tests, rats were anesthetized and decapitated. The brains were removed immediately. Then, the cortex and hippocampus were dissected and homogenized in 9 volumes of cold saline using a glass homogenizer. The homogenates were centrifuged at 3000 ×g for 10 min at 4°C, and the supernatants were used for biochemical determinations. Blood samples were collected in 2 mL Eppendorf tubes and centrifuged at 4000 ×g for 10 min at 4°C to separate the serum from whole blood. The serum samples were stored at −80°C until assay.

### 2.7. Determination of Acetylcholine Esterase (AChE) and Choline Acetyltransferase (ChAT) Activity

The experiments were performed according to the instructions of the AChE and ChAT activity kits (Nanjing Jiancheng Bioengineering Institute, China). Activity was expressed in units per gram of input protein. Protein concentration was determined using the bicinchoninic acid (BCA) assay.

### 2.8. Measurement of Superoxide Dismutase (SOD), Catalase (CAT) Activity, and MDA Levels

Commercial kits (Nanjing Jiancheng Bioengineering Institute, China) were employed to assess the activity of SOD and CAT, as well as the MDA level. The activity of SOD and CAT was expressed as units/mg protein. Brain MDA content was expressed as nmol/mg protein. The protein concentration was estimated using a BCA kit.

### 2.9. Estimation of ROS and 8-Hydroxy-2-deoxyguanosine (8-OHdG) Concentrations

The ROS concentrations in hippocampus were measured using the 2,7-dichlorodihydrofluorescein diacetate (DCF-DA). Crude homogenate extracts of 100 mg hippocampus tissue were taken for HU exposed and control animals. The extracts were incubated with 160 *μ*L of 10 *μ*M DCF-DA mixture for 4 h at 37°C in a dark environment. The fluorescent product formed in DCF-DA was quantified using a fluorescence microplate reader at the excitation and emission wavelengths of 485 and 525 nm, respectively. The ROS in serum and 8-OHdG concentrations were measured using ELISA kits (R&D, USA), according to the protocol of manufacturer. To summarise, samples or standards were added to the assay plate, which was precoated with ROS or 8-OHdG, and incubated at 37°C for 1 h. Then the assay plate was washed. Next, HRP-conjugated secondary antibody was added. After incubation and washing, the enzyme substrate was added. The samples were gently mixed and incubated for 15 min at 37°C in the dark. Finally, stop solution was added and the absorbance (450 nm) was examined within 15 min. The concentration of ROS or 8-OHdG in the samples was then determined by comparing the OD of the samples to the standard curve.

### 2.10. Statistical Analysis

All data were shown as mean ± SEM (standard error of the mean). Data were analyzed statistically using the unpaired Student's *t*-test, two-tailed (for 2 groups), and one-way ANOVA with Tukey-Kramer post hoc correction (for more-than-2 groups), unless stated otherwise. The criterion for a significant difference was ^*∗*^*P* < 0.05 and ^*∗∗*^*P* < 0.01.

## 3. Results

### 3.1. Effects of HU on Spatial Memory in MWM Test

**Figure 2:**
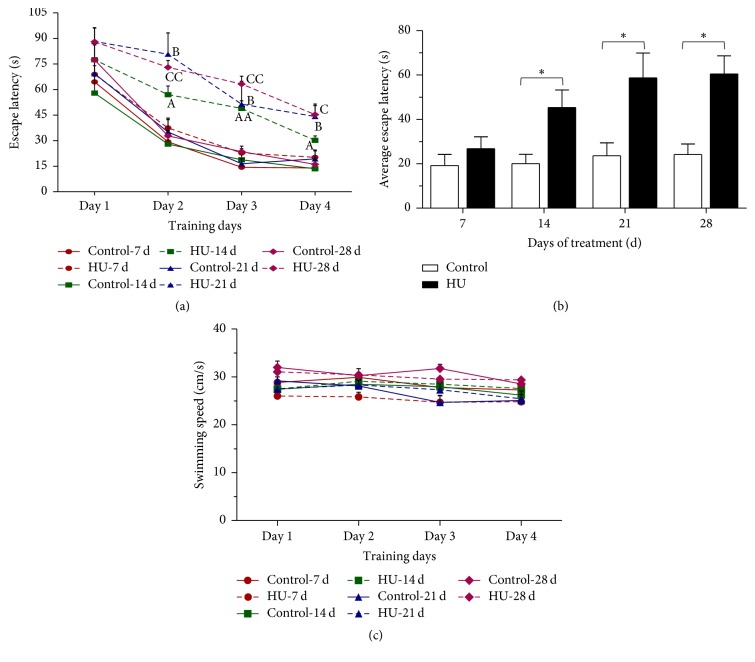
Effects of HU (hindlimb unloading) on the acquisition trials of the MWM (Morris water maze) test in rats. (a) Escape latency of each training day, (b) average escape latency in each group, and (c) swimming speed. Values represent mean ± SEM. ^A^*P* < 0.05 and ^AA^*P* < 0.01, compared with Control-14 d; ^B^*P* < 0.05, compared with Control-21 d; ^C^*P* < 0.05 and ^CC^*P* < 0.01, compared with Control-28 d; ^*∗*^*P* < 0.05, compared with time-matched control.

For the memory acquisition, there was a decrease of latency to find the platform over training days for all groups. In the 14/21/28 days' treatment groups, HU rats exhibited significantly longer escape latencies from day 2 to day 4 (acquisition phase) compared with the time-matched controls (*P* < 0.05 or *P* < 0.01). However, the HU-7 d rats spent the same time as Control-7 d to find the platform (Figure 2(a)). Average escape latency (the average latency of four training days) in each group was calculated to estimate the effect of various HU duration on memory. The four control groups showed comparable average escape latencies to the platform, and there was no significant difference between Control-7 d and HU-7 d. While compared with HU-7 d, the average escape latency of HU-14 d, HU-21 d, and HU-28 d was extended by 69.1%, 119.1%, and 125.7%, respectively (Figure 2(b)).

**Figure 3:**
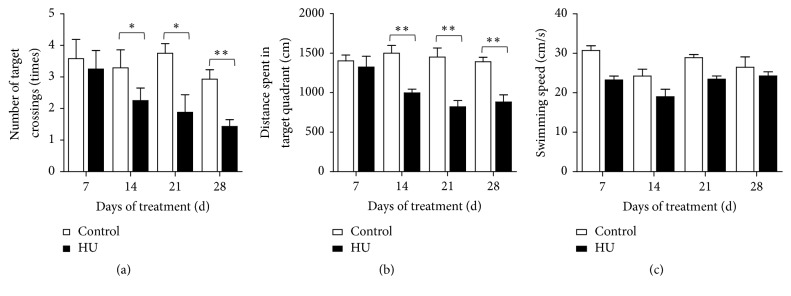
Effects of HU (hindlimb unloading) on the probe trial of the MWM (Morris water maze) test in rats. (a) Number of target crossings, (b) distance spent in target quadrant, and (c) swimming speed. Values represent mean ± SEM. ^*∗*^*P* < 0.05 and ^*∗∗*^*P* < 0.01, compared with time-matched control.

For the probe trial, HU rats performed fewer crossings and swam less in the target quadrant than control in the 14/21/28 days' treatment groups (*P* < 0.05 or *P* < 0.01). The four control groups had similar target crossings and distance of target quadrant. In comparison with HU-7 d, the number of target crossings was decreased by 30.8%, 42.2%, and 56.0% in the HU-14 d, HU-21 d, and HU-28 d-groups; moreover, the distance of target quadrant declined by 24.6%, 37.9%, and 33.4% (Figures 3(a) and 3(b)).

There were no significant changes in the swimming speed among the groups throughout the experiments (Figures 2(c) and 3(c)), confirming that any change in water maze performance was not due to differences in motor activity and swimming ability.

### 3.2. Effects of HU on Cognitive Function in the Shuttle Box Test

**Figure 4:**
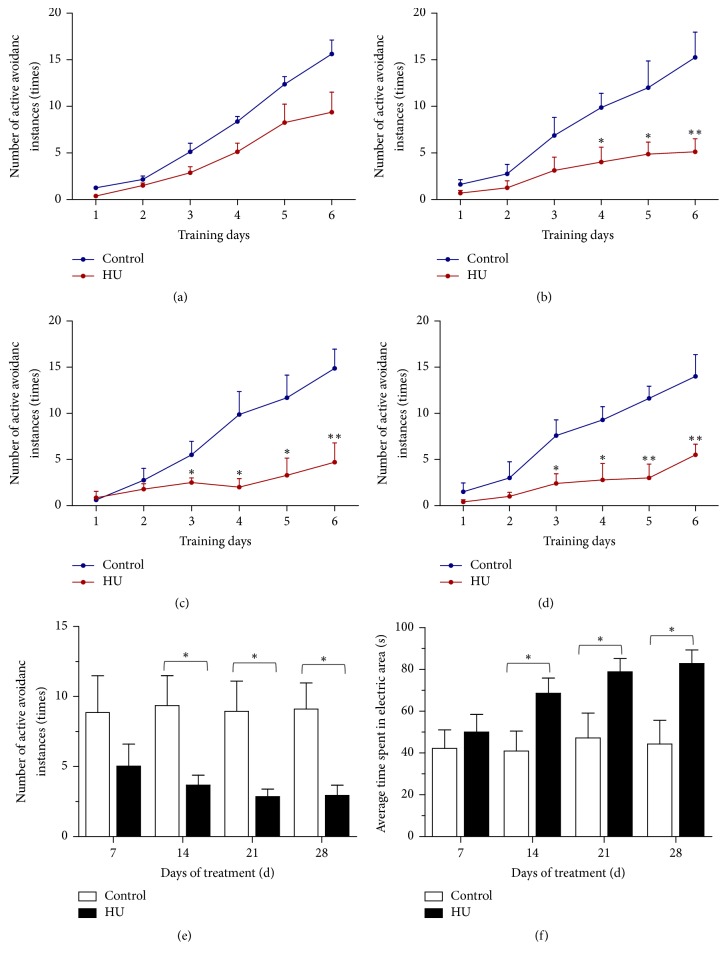
Effects of HU (hindlimb unloading) on cognitive function of rats in the shuttle box test. (a)–(d) Number of active avoidance instances on each training day for 7 d-groups, 14 d-groups, 21 d-groups, and 28 d-groups, respectively. (e) Average number of active avoidance instances in each group. (f) Average time spent in electric area in each group. Values represent mean ± SEM. ^*∗*^*P* < 0.05 and ^*∗∗*^*P* < 0.01, compared with time-matched control.

In this task, active avoidance behaviors in responses represent learning and memory ability.  Figure 4 shows the performance of rats in the shuttle box test.The number of active avoidance instances was increased over the course of the test in all groups. Compared with control, HU rats performed more poorly for avoiding the foot shock actively. There were no differences in 7 d-groups, while a difference appeared as a stable consecutive significance from day 3 to day 6 (*P* < 0.05 or *P* < 0.01) in 14/21/28 d-groups (Figures 4(a)-4(d)). Average number of active avoidance instances and time spent in electric area in each group were also calculated. These two indexes in the four control groups were similar. In comparison with HU-7 d, the average number of active avoidance instances for HU-14 d, HU-21 d, and HU-28 d dropped by 26.8%, 43.1%, and 41.5%, respectively (Figure 4(e)); on the contrary, the average time spent in electric area was increased by 37.1%, 57.6%, and 65.7% (Figure 4(f)).

### 3.3. Effects of HU on AChE and ChAT Activity in Hippocampus and Cortex

**Figure 5:**
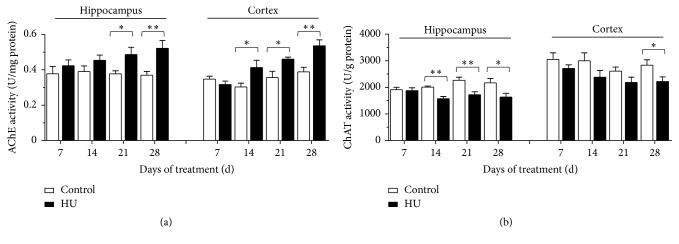
Effects of HU (hindlimb unloading) on the activities of AChE and ChAT in rat brain tissues. (a) AChE activity and (b) ChAT activity. Values represent mean ± SEM. ^*∗*^*P* < 0.05 and ^*∗∗*^*P* < 0.01, compared with time-matched control.

The AChE activity of hippocampus and cortex was shown in Figure 5(a). After 21 d or 28 d HU, AChE activity in hippocampus was significantly enhanced compared with the time-matched controls (*P* < 0.05 and *P* < 0.01); in comparison with HU-7 d, the AChE activity of hippocampus for HU-14 d, HU-21 d, and HU-28 d was elevated by 7.2%, 14.8%, and 23.1%, respectively. In cortex, AChE activity of HU rats increased noticeably compared with the time-matched controls in 14/21/28 d-groups (*P* < 0.05, *P* < 0.05, and *P* < 0.01); the rats of HU-14 d, HU-21 d, and HU-28 d had better AChE activity than HU-7 d (30.6%, 45.4%, and 69.4%, resp.).

Figure 5(b) exhibited the ChAT activity of hippocampus and cortex after different duration of HU. After 14 d, 21 d, and 28 d HU, ChAT activity in hippocampus was reduced significantly compared with the time-matched controls (*P* < 0.05, *P* < 0.01, and *P* < 0.01); in comparison with HU-7 d, the ChAT activity of hippocampus for HU-14 d, HU-21 d, and HU-28 d was decreased by 16.3%, 8.3%, and 12.9%, respectively. In cortex, ChAT activity of HU-28 d rats was attenuated notably compared with the control-28 d (*P* < 0.05); the rats of HU-14 d, HU-21 d, and HU-28 d had weaker ChAT activity than HU-7 d (12.1%, 19.4%, and 18.0%, resp.).

### 3.4. Effects of HU on the Activity of SOD, CAT, and MDA Level in Rat Hippocampus

**Figure 6:**
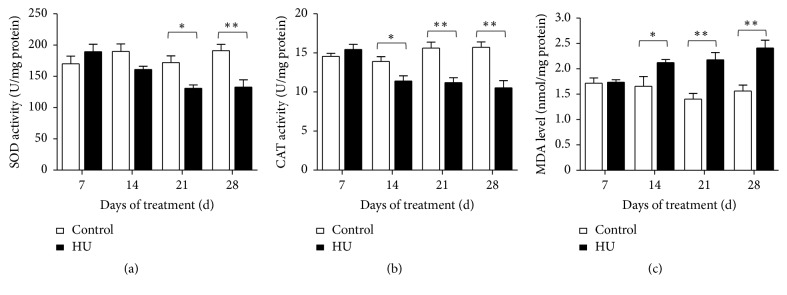
Effects of HU (hindlimb unloading) on the activities of SOD, CAT, and MDA level in rat hippocampus. (a) SOD activity, (b) CAT activity, and (c) MDA level. Values represent mean ± SEM. ^*∗*^*P* < 0.05 and ^*∗∗*^*P* < 0.01, compared with time-matched control.

As shown in Figure 6, effects of HU on the cerebral antioxidant system were depicted. The results demonstrated that HU leads to considerable decline for SOD activity in 21 d and 28 d treatment groups (*P* < 0.05 and *P* < 0.01). In comparison with HU-7 d, the SOD activity for HU-14 d, HU-21 d, and HU-28 d was lowered by 15.1%, 30.9%, and 30.0%, respectively (Figure 6(a)). Similar to SOD, activity decrease appeared on CAT after HU. Compared to time-matched controls, there were significant differences in HU-14 d, HU-21 d, and HU-28 d (*P* < 0.05, *P* < 0.01, and *P* < 0.01). Compared with HU-7 d, the CAT activity for HU-14 d, HU-21 d, and HU-28 d was reduced by 26.1%, 27.4%, and 31.7%, respectively (Figure 6(b)). Nevertheless, the MDA level was elevated remarkably in HU-14 d, HU-21 d, and HU-28 d compared with the time-matched controls (*P* < 0.05, *P* < 0.01, and *P* < 0.01). In comparison with HU-7 d, the MDA level for HU-14 d, HU-21 d, and HU-28 d was increased by 22.3%, 25.4%, and 38.8%, respectively (Figure 6(c)).

### 3.5. Effects of HU on ROS and 8-OHdG Concentrations in Rat Hippocampus

**Figure 7:**
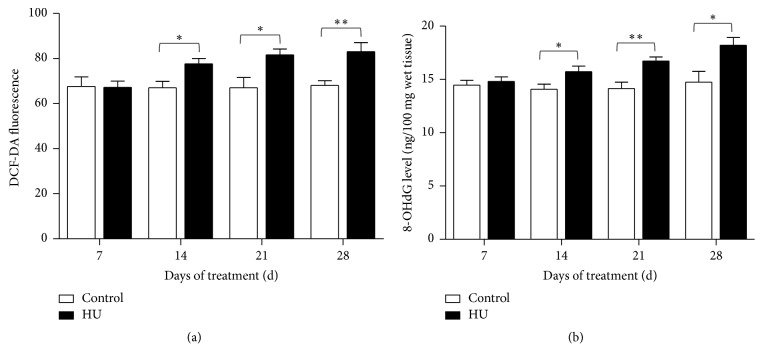
Effects of HU (hindlimb unloading) on ROS and 8-OHdG level in rat hippocampus. (a) DCF-DA fluorescence and (b) 8-OHdG level. Values represent mean ± SEM. ^*∗*^*P* < 0.05 and ^*∗∗*^*P* < 0.01, compared with time-matched control.

The results showed that ROS and 8-OHdG concentrations in hippocampus were increased for HU rats. For ROS, the increase was evident in HU-14 d, HU-21 d, and HU-28 d compared with the time-matched controls (*P* < 0.05, *P* < 0.05, and *P* < 0.01). In comparison with HU-7 d, the ROS concentrations for HU-14 d, HU-21 d, and HU-28 d were increased by 15.5%, 21.4%, and 23.5%, respectively (Figure 7(a)). For 8-OHdG, the increase was apparent in HU-14 d, HU-21 d, and HU-28 d compared with the corresponding controls (*P* < 0.05, *P* < 0.01, and *P* < 0.05), indicating that oxidative DNA damage increased. The rats of HU-14 d, HU-21 d, and HU-28 d had higher 8-OHdG concentrations than HU-7 d (6.2%, 13.0%, and 23.0%, resp., Figure 7(b)).

### 3.6. Effects of HU on ROS and 8-OHdG Concentrations in Rat Serum

**Figure 8:**
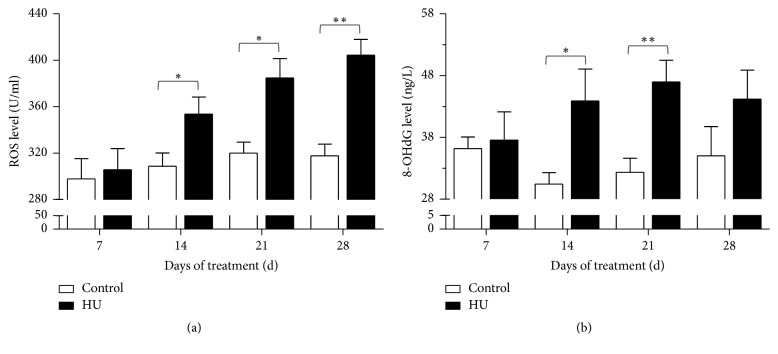
Effects of HU (hindlimb unloading) on ROS and 8-OHdG level in rat serum. (a) ROS level and (b) 8-OHdG level. Values represent mean ± SEM. ^*∗*^*P* < 0.05 and ^*∗∗*^*P* < 0.01, compared with time-matched control.

The results showed that ROS and 8-OHdG concentrations in serum were raised for HU rats. For ROS, the increase was evident in HU-14 d, HU-21 d, and HU-28 d compared with the time-matched controls (*P* < 0.05, *P* < 0.05, and *P* < 0.01). In comparison with HU-7 d, the ROS concentrations for HU-14 d, HU-21 d, and HU-28 d were increased by 15.7%, 25.8%, and 32.3%, respectively (Figure 8(a)). For 8-OHdG, the increase was apparent in HU-14 d and HU-21 d compared with the corresponding controls (*P* < 0.05 and *P* < 0.01), indicating that oxidative DNA damage increased. The rats of HU-14 d, HU-21 d, and HU-28 d had higher 8-OHdG concentrations than HU-7 d (16.9%, 25.0%, and 17.6%, resp., Figure 8(b)).

### 3.7. Effects of Different HU Duration on Behavioral and Biochemical Parameters

To fully reveal the dynamic HU effects, the variation tendencies of behavioral and biochemical parameters for different HU duration were depicted (Table 1). When we compared longer HU exposure groups with HU-7 d, differential variation tendencies of parameters were presented. For behavioral tests, some parameters of HU rats were increased or decreased continuously from 7 d to 28 d. For instance, escape latency and time spent in electric area were increased all the time, whereas the number of target crossings for rats was decreased. Relative to HU-7 d, although the distance spent in target quadrant and number of active avoidance instances for HU-14 d, HU-21 d, and HU-28 d all lowered, the biggest drop was for HU-21 d. This suggested that diverse indexes in the same cognitive task had different sensitivity to HU, and the HU effect on learning and memory was complicated and various.

For biochemical tests, the same situation also existed. For example, AChE activity and MDA and ROS levels for HU rats were increased all the time from 7 d to 28 d. On the contrary, SOD and CAT activity were decreased continuously from 7 d to 28 d. Though the ChAT activity for HU-14 d, HU-21 d, and HU-28 d all decreased relative to HU-7 d, the smallest drop was for HU-21 d in hippocampus. In addition, the biggest increase in 8-OHdG of serum was for HU-21 d relative to HU-7 d. The differential variation suggested the complexity of the regulation for cholinergic system and redox balance in HU rats and suggested that simulated microgravity effects may function by multiple other ways, so the cholinergic regulation and oxidative stress under simulated microgravity were different from other conditions.

## 4. Discussion

This paper attempted to shed light on the effects of HU on learning and memory and how the memory function varies with the extended HU duration. HU in this study, as a model to reproduce the chronic weightless bearing, has been used in many laboratories around the world [21]. This model results in a cephalad fluid shift and avoids weight-bearing for the hindquarters. From cardiovascular, muscular, and hormonal points of view, this condition has been considered to be very similar to microgravity in space [24]. Based on this, lots of research were carried out [25–28]. One similarity of these studies was that all of them focused on the simulated weightlessness effect for only one single period. To reflect the dynamic change for memory function in rats over a longer duration of simulated weightlessness, this study investigated four different periods to HU exposure.

Behavioral testing is an important approach to assess the learning and memory abilities. Morris water maze is generally accepted as an indicator of spatial learning and reference memory. It is a reliable and convenient method to assess hippocampal-dependent cognitive function in rodents, because this task is relatively insensitive to differences in body weight and appetite and does not require food or water restriction and learning in the water maze proceeds rapidly and efficiently. The present study demonstrated that exposure to HU for more than 14 d led to spatial memory deficits in rats, as indicated by increases in escape latency and decreases in the number of target crossings and the distance in target quadrant of the MWM test.

To identify whether the HU effects on memory were only for a specific task, we adopted another method for cognitive evaluation. Shuttle box test, a signaled two-way active avoidance task, is one of the most widely used instrumental conditioning paradigms [29]. In this task, animals had to learn to predict the occurrence of an aversive event (shock) based on the presentation of a specific stimulus (tone and/or light), in order to avoid the aversive event by moving to a different compartment. It is a complex conditioned reflex task involving different forms of learning, as well as different stages of the acquisition process. It not only represents an operant conditioning paradigm, but also exhibits a form of associative learning [30]. In our study, the number of active avoidance instances had a significant decline for longer-than-14 d HU rats, while the time spent in electric area increased remarkably. The inferior active avoidance behaviors indicated that HU contributed to the memory deficits in active avoidance task.

In a word, HU exposure reduced the learning and memory ability of rats in different tasks. This was in accordance with previous results [27, 31]. Indeed, microgravity ground simulation studies provided some evidences to the hypothesis of a microgravity-induced cognitive impairment. A previous study showed that two genes (Grin 1 and Itga 3), involved in learning and memory, were significantly altered in the brains of 2-week HU mice [32].

Studies have shown that cognitive functioning is closely related to the central cholinergic system [33]. The neurotransmitter ACh, which is synthesized by ChAT and hydrolyzed by AChE, plays a vital role in central and peripheral control of multiple cognitive processes including learning and memory [34]. A*β*-induced amnesia can be reversed by elevating the central cholinergic activity through various pharmacological manipulations in rats [35]. Notably, inhibition of AChE is currently the most common treatment strategy for the symptoms of Alzheimer disease (AD) [36]. Additionally, it is reported that the degree of reduction of cerebral ChAT activity is significantly correlated with the severity of dementia [37]. In our results, there was a significant increase of AChE activity in both hippocampus (HU-21 d and HU-28 d) and cortex (HU-14 d, HU-21 d, and HU-28 d); simultaneously, the ChAT activity was significantly decreased not only in hippocampus (HU-14 d, HU-21 d, and HU-28 d) but also in cortex (HU-28 d). These findings indicated the dysfunction of cholinergic system was induced by HU. Based on the results, ChAT activity of cortex response to HU was not as sensitive as that of hippocampus. This reflected the differential response of the same enzyme to HU in different brain regions.

Accumulating studies have substantiated that oxidative stress can cause learning and memory impairment [38, 39]. Learning and memory function is primarily governed by the hippocampus, which is markedly susceptible to oxidative stress. In our study, longer-than-14 d HU exposure caused significant changes in oxidative stress markers. The antioxidant enzyme activity, including SOD and CAT, was reduced. The reduction in the antioxidant defense mechanisms increased the oxidative stress in the hippocampus and provided a reasonable explanation for the memory deficits accompanying HU exposure. Measurements of MDA level provide a convenient index of lipid peroxidation. In this study, MDA level of hippocampus was increased significantly by longer-than-14 d HU exposure. In line with this, lipid peroxidation in rat brain was aggravated due to the 14 d-simulated weightlessness [40]. Our results also demonstrated that HU exposure increases the oxidative stress by elevating ROS and 8-OHdG concentrations in rat serum. 8-OHdG is produced via the oxidative damage of DNA by reactive oxygen and nitrogen species and also serves as an established marker of oxidative stress. To sum up, HU exposure produced oxidative damage as evidenced by significant increase in MDA levels and ROS and 8-OHdG concentrations and decrease in antioxidant enzymes activity. Long-term exposure to oxidative stress and DNA damage may result in neuronal injury. In previous studies, cognitive deterioration was associated with elevation of MDA level in aged female rats [41]. Increased oxidative DNA damage was accompanied by spatial memory deficit in chronic intermittent hypoxia rats [42]. In a posttraumatic stress disorder (PTSD) model, rats displayed the hippocampus-dependent spatial memory deficit accompanied by the upregulation of NOX2 and 8-OHdG [43]. All the above alterations suggested that both central and peripheral oxidative damage are potential contributors to the cognitive impairments that are associated with HU. Notably, SOD activity for HU-14 d was not significantly reduced like HU-21 d and HU-28 d. Similarly, 8-OHdG concentrations for HU-28 d were not evidently increased like HU-14 d and HU-21 d. These suggested that oxidative damage induced by HU was a complex business.

According to the results of behavioral and biochemical tests, there were minor changes but not significant in all parameters between the control and HU rats after 7 d of HU. After 14 d of HU, either behavioral or biochemical parameters had dramatic changes, as manifested by deteriorated cognitive, cholinergic function and increased oxidative damage. After 21 d and 28 d of HU, the situation seemed to be getting worse. Given that the 7-day observation interval is too long, some changes in the process may be covered; besides, the effect of longer HU exposure (more than 28 d) was not clear; future research will shorten the observation interval and prolong the HU treatment.

Taken together, by a combination of behavioral and biochemical experiments, this study demonstrates that simulated weightlessness of more than 14 d-duration HU could damage the ability of learning and memory in rats. Consistently, this effect was accompanied by a significant increase in cholinergic dysfunction and oxidative stress, although some indicators had their own characteristics of change. The decline of cognitive functions may be a complex gradual process and comprehends a large variety of molecular alterations, but cholinergic dysfunction and oxidative damage at least in part contribute to this process. These results will not only give the readers a better understanding of the simulated microgravity effects on learning and memory, but also hold promise for effective countermeasures development about memory deficits under extreme environments by present possible biochemical targets.

## Figures and Tables

**Table 1:** The variation tendencies of behavioral and biochemical parameters for different HU (hindlimb unloading) duration.

Parameters		HU-7 d	HU-14 d	HU-21 d	HU-28 d
Behavioral parameters					
Average escape latency (s)		0	69.1% ↑	119.1% ↑	125.7% ↑
Number of target crossings (times)		0	30.8% ↓	42.2% ↓	56.0% ↓
Distance spent in target quadrant (cm)		0	24.6% ↓	37.9% ↓	33.4% ↓
Average number of active avoidance instances (times)		0	26.8% ↓	43.1% ↓	41.5% ↓
Average time spent in electric area (s)		0	37.1% ↑	57.6% ↑	65.7% ↑
Biochemical parameters					
AChE activity (U/mg protein)	(hippocampus)	0	7.2 % ↑	14.8% ↑	23.1% ↑
	(cortex)	0	30.6% ↑	45.4% ↑	69.4% ↑
ChAT activity (U/g protein)	(hippocampus)	0	16.3% ↓	8.3% ↓	12.9% ↓
	(cortex)	0	12.1% ↓	19.4% ↓	18.0% ↓
SOD activity (U/mg protein)	(hippocampus)	0	15.1% ↓	30.9% ↓	30.0% ↓
CAT activity (U/mg protein)		0	26.1% ↓	27.4% ↓	31.7% ↓
MDA level (nmol/mg protein)		0	22.3% ↑	25.4% ↑	38.8% ↑
ROS (DCF-DA fluorescence)	(hippocampus)	0	15.5% ↑	21.4% ↑	23.5% ↑
8-OHdG (ng/100 mg wet tissue)		0	6.2% ↑	13.0% ↑	23.0% ↑
ROS (U/ml)	(serum)	0	15.7% ↑	25.8% ↑	32.3% ↑
8-OHdG (ng/L)		0	16.9% ↑	25.0% ↑	17.6% ↑

*Note.* Numbers represent the change percentage of parameters for HU-14 d, HU-21 d, and HU-28 d compared to those of HU-7 d. “↑” indicates increase; “↓” indicates decrease.
